# Dietary replacement of whole soybean grain with micronized defatted soybean meal in dairy cow diets: effects on intake, digestibility, ruminal parameters, and milk yield and composition

**DOI:** 10.1007/s11250-026-05134-2

**Published:** 2026-06-12

**Authors:** Thiago Thiarles Braw André Furlan Romualdo Angelo Abraãn Lincon Picciuto Maciel, Eliane Sayuri Miyagi Okada, Euclides Reuter De Oliveira, Jefferson Rodrigues Gandra, Vera Lúcia Banys, Thaís Da Silva Fernandes, Andréa Maria de Araújo Gabriel, Claudia Andrea Lima Cardoso, Vicente Ribeiro Rocha Júnior, Ranney Késia Oliveira de Jesus Silva, Flávio Pinto Monção

**Affiliations:** 1 College of Agricultural Sciences, Federal University of Great Dourados, Rod. Dourados-Itahum, Km 12, Dourados, Mato Grosso do Sul, Brazil; 2 School of Veterinary and Animal Science, Federal University of Goiás, Rodovia Goiânia/Nova Veneza (GO 462), km 0, Campus Samambaia (Campus II), Goiânia, Goiás, Brazil; 3 College of Veterinary Medicine, Federal University of Southern and Southeastern Pará, R. Alberto Santos Dumont, Xinguara, PA Brazil; 4 Department of Agricultural Sciences, Federal University of Jataí, Rua Riachuelo-Setor-Samuel Grahan, Goiás, Brazil; 5 Department of Agricultural Sciences, State University of Montes Claros, Avenida Reinaldo Viana, Janaúba, Minas Gerais, 2630 Brazil

**Keywords:** Nutrient intake, Animal performance, Digestion, Milk yield

## Abstract

This study aimed to evaluate effects of replacing whole soybean grain with micronized defatted soybean meal (MDSM) in diets of lactating dairy cows on performance, digestion, metabolism, and milk yield and composition. Ten primiparous Jersey cows with an initial body weight (BW) of 410 ± 9 kg and 105 ± 5 days in milk were used. Five replacement levels (0, 25, 50, 75, and 100% of dietary DM) of whole soybean grain with MDSM were evaluated. The experiment was conducted as two simultaneous 5×5 Latin squares, each composed of five animals, five treatments, and five experimental periods, totaling 80 days. Each period lasted 16 days, including 12 days for adaptation and 4 days for data collection. No differences were observed among experimental diets for dry matter intake (mean = 14.58 kg day^-1^; p = 0.08) or organic matter intake (mean = 13.60 kg day^-1^; p = 0.08). Increasing dietary inclusion of MDSM resulted in greater crude protein intake, with the highest intake observed at 14% MDSM inclusion. A linear increase (P < 0.05) was observed in digestibility of dry matter, organic matter, crude protein, ether extract, and starch as MDSM inclusion increased. The inclusion level that maximized short-chain fatty acid production was 9.22%. Milk yield corrected to 3.5% fat increased linearly with increasing MDSM inclusion. Inclusion of MDSM did not affect milk fat (p = 0.41), protein (p = 0.86), or lactose (p = 0.88) concentrations. Complete replacement of whole soybean grain with MDSM in diets of Jersey cows increased milk yield, improved feed efficiency, and did not alter milk composition. Additionally, it enhanced digestibility of dry matter, organic matter, crude protein, and starch.

## Introduction

Dairy farming plays a vital role in Brazil’s social and economic context. Approximately 35 billion liters of milk are produced annually (Anuário do Leite 2024), with up to 80% of this volume originating from crossbred Holstein × Zebu cows. Feeding management is one of the main factors defining production systems. In general, forage plants represent the primary nutrient source for dairy cattle during the rainy season, characterizing extensive production systems. However, under tropical climate conditions, rapid fluctuations in forage availability and nutritional quality occur, requiring supplementation with concentrate feeds in dairy cow diets (Monção et al. 2020, Santana et al. 2019; Cordeiro et al. 2023; Leal et al. 2023).

In intensive production systems, higher inclusion of protein and energy concentrates is commonly adopted, which may increase milk production costs (Durães et al. 2024). Among dietary components, protein sources represent one of the major cost drivers, and soybean products, either as whole grain or processed forms, are traditionally used for this purpose (Marques et al. 2024). Soybeans provide an important source of nitrogenous compounds for dairy cows; however, their use may be limited by high crude fat content and presence of antinutritional factors, such as urease activity. Soybean meals have been widely used as a protein source in ruminant diets, and, despite their high nutritional value and absence of major limiting factors, this is associated with high acquisition costs.

Defatted soybean meal is considered one of the most valuable protein sources for ruminants, due to its favorable amino acid profile compared with alternative protein feeds. Nevertheless, improvements in animal feed efficiency are not always achieved, partly because of substantial variation in particle size observed in conventional soybean meals and whole soybean grain. Micronization of defatted soybean meals stands for a processing strategy that may enhance nutrient availability compared with ground soybean grain and conventional soybean meals. This process consists of ultrafine grinding using compressed-air mills and sieves ranging from 1 to 10 µm, producing a fine yellowish powder. Micronization is expected to increase nutrient availability and digestibility within the gastrointestinal tract.

Despite these potential advantages, information about the use of micronized defatted soybean meals in ruminant diets is still scarce. In diets for lactating cows, knowledge gaps persist concerning effects of different inclusion levels of MDSM on nutrient intake, digestibility, and animal performance. Therefore, optimal inclusion level of micronized defatted soybean meal can replace traditional protein sources such as whole soybean grain, improving feed efficiency and milk composition.

This study aimed to evaluate partial replacement of whole soybean grain with MDSM in diets of lactating dairy cows and its effects on intake, nutrient digestibility, ruminal parameters, microbial protein synthesis, and milk production and composition.

## Material and methods

### Ethical approval, site, and animal management

All experimental procedures were approved by the Animal Care and Use Committee, following guidelines of the Committee on Ethics in Animal Use (Protocol No. 33/2020). The experiment was conducted at the experimental farm of Universidade Federal da Grande Dourados (UFGD), located in Mato Grosso do Sul, Brazil.

Ten Jersey cows with an initial body weight (BW) of 410 ± 9 kg, 105 ± 5 days in milk, an average milk yield of 15 ± 2 kg day^−^^1^ at the beginning of the experiment, and a mean age of 60 months were used. Cows were housed in individual stalls (21 m^2^) equipped with feed troughs and waterers.

### Diets and management

Five experimental diets were evaluated: Diet 1 (Control), consisting of the basal diet without micronized defatted soybean meal (MDSM); Diet 2, with 25% replacement of whole soybean grain with MDSM; Diet 3, with 50% replacement; Diet 4, with 75% replacement; and Diet 5, with 100% replacement of whole soybean grain with MDSM.

The experiment was conducted using two simultaneous 5 × 5 Latin squares, each composed of five animals, five treatments, and five experimental periods, totaling 80 days. Each experimental period lasted 16 days, including 12 days for adaptation to diets and management conditions and 4 days for data collection and sampling.

Diets were formulated according to NASEM (2021) recommendations to be isonitrogenous, containing 155 g kg^−^^1^ crude protein (CP) on a dry matter basis. Cows were fed twice daily as a total mixed ration (TMR) at 0730 and 1430 h. The forage-to-concentrate ratio, expressed on a dry matter basis, was 51:49.

### Chemical analysis

During the last four days of each experimental period, samples of offered feed, refusals, and feces were collected in the morning and stored at − 20 °C until analysis. Representative subsamples of feed ingredients, refusals, and fecal material were dried in a forced-air circulation oven at 55 °C for 72 h and ground using a Wiley mill to pass through a 1-mm screen.

Samples were analyzed for dry matter (DM; Method No. 950.05), ash (Method No. 942.05), crude protein (CP; *N* × 6.25; Kjeldahl method, Method No. 984.13), and ether extract (EE; Method No. 920.39) according to procedures described by the Association of Official Analytical Chemists (AOAC] Association of Official Analytical Chemists International 2005) (Table 1). Organic matter (OM) was calculated as the difference between DM and ash content.

**Table 1:** Ingredients and chemical composition of experimental diets

Ingredient	Micronized defatted soybean meal (g/kg)				
	0	25	50	75	100
Whole-plant corn silage	410	410	410	410	410
Tifton 85 hay	100	100	100	100	100
Ground corn	220	220	220	220	220
Whole soybean^1^	240	180	120	60	0
MDSM^2^	0	60	120	180	240
Mineral mix^3^	30	30	30	30	30
	Composition (g kg^−1^)				
Dry matter	606	605	604	603	602
Organic matter	921	921	920	919	919
Crude protein	154	157	159	162	165
Neutral detergent fiber	357	356	355	353	352
Neutral detergent fiber, ash- and protein-corrected	209	265	259	248	234
Starch	291	291	290	289	288
Ether extract	65.5	57.8	50.1	42.3	34.6
Acid detergent fiber	212	213	214	215.	216
Lignin	40.9	39.6	38.2	36.8	35.4
Nonfiber carbohydrates	344	350	356	362	367
Total digestible nutrients^4^	659	649	640	630	620
Net energy for lactation^5^ (Mcal kg^−1^)	1.50	1.47	1.45	1.42	1.40

^1^ Whole soybean grain (crude protein, 380 g kg^−^^1^; ether extract, 200 g kg^−^^1^; neutral detergent fiber, 210 g kg^−^^1^; cost per metric ton = US$ 330.00)

^2^ Micronized defatted soybean meal (crude protein, 410 g kg^−^^1^; ether extract, 60 g kg^−^^1^; neutral detergent fiber, 230 g kg^−^^1^; cost per metric ton = R$ 4,410.00 or US$ 843.21; exchange rate: 1 US$ = R$ 5.23)

^3^ Mineral mix composition: Ca, 110 g kg^−1^; P, 42 g kg^−^^1^; S, 18 g kg^−^^1^; Mg, 20 g kg^−^^1^; Na, 123 g kg^−^^1^; Co, 14 mg kg^−^^1^; Cu, 600 mg kg^−^^1^; Cr, 20 mg kg^−^^1^; Fe, 1,050 mg kg^−^^1^; I, 28 mg kg^−^^1^; Mn, 2,000 mg kg^−^^1^; Se, 18 mg kg^−^^1^; Zn, 2,800 mg kg^−^^1^; biotin, 80 mg kg^−^^1^; vitamin A, 240,000 IU kg^−^^1^; vitamin D, 100,000 IU kg^−^^1^; vitamin E, 100,000 IU kg^−^^1^; monensin sodium, 600 mg kg^−^^1^

^4,5^ Calculated according to NRC, National (2001)

Neutral detergent fiber (NDF) and acid detergent fiber (ADF) concentrations were determined according to Van Soest et al. (1991). Lignin concentration was determined following AOAC] Association of Official Analytical Chemists (2012) Method 973.18, in which ADF residue was treated with 72% sulfuric acid. Non-fiber carbohydrates (NFC) were calculated according to Hall (2000), and total digestible nutrients (TDN) of ingredients were estimated according to NRC, National (2001).

Starch concentration was determined using the method described by Hendrix (1993). Absorbance readings were obtained using commercial enzymatic colorimetric kits analyzed with an automatic biochemical analyzer.

### Particle selection, feed intake, and nutrient digestibility

For evaluation of fecal feed residues, fecal samples (500 g) were collected on day 15 directly from the rectal ampulla of each cow. Samples were then washed through a 1-mm sieve to retain undigested feed particles. The retained fecal residues were dried in a forced-air circulation oven at 55 °C for 72 h and ground using a mill equipped with a 1-mm screen for later dry matter chemical analysis.

During each experimental period (days 12 to 16), samples of feed refusals from each cow were analyzed for particle size distribution using a Penn State Particle Separator (Nasco, Fort Atkinson, WI, USA) equipped with stratified sieves.

From days 12 to 16 of each experimental period, forage and concentrate offered, as well as refusals from each cow, were weighed daily to determine feed intake, allowing refusals equivalent to 5% to 10% of feed offered (as-fed basis). Amounts of feed offered and refusals were recorded daily for each animal to calculate individual feed intake. In addition, diet and refusal samples from each cow were collected every three days throughout the experimental period.

Apparent total-tract nutrient digestibility was determined on day 13 of each experimental period using the total fecal collection method over 24 h. Feces were collected after spontaneous defecation, placed in plastic containers, and stored at − 20 °C. At the end of each collection period, total fecal output was weighed after homogenization. Digestibility coefficients (DC) were calculated as follows: DC = [(kg ingested − kg excreted)/(kg ingested)] × 100.

### Ruminal analysis

On the 16th day of each experimental period, ruminal digesta samples were collected using an esophageal probe approximately 4 h after the morning feeding. Samples were filtered through cheesecloth to obtain ruminal fluid, and ruminal pH was measured immediately after collection using a pH meter (MB-10, Marte, Santa Rita do Sapucaí, Brazil) in a 100-mL subsample.

Subsequently, 20 mL of ruminal fluid was transferred to sealed plastic containers and immediately stored at − 20 °C for later analysis. Volatile fatty acid (VFA) concentrations were determined using a high-performance liquid chromatograph (Hewlett Packard 5890 Series II GC) equipped with a packed Carbopack column (3 m), coupled to an ultraviolet detector (Hewlett Packard 3396 Series II Integrator) and an injector system (Hewlett Packard 6890 Series).

### Nitrogen balance and microbial protein synthesis

On days 12 and 16 of each experimental period, urine samples were collected during spontaneous urination approximately 4 h after feeding. At the end of the sampling period, composite samples were prepared for each animal. Urine pH was adjusted, when necessary, to values below 3.0 to prevent bacterial degradation of purine derivatives and precipitation of uric acid.

Concentrations of purine derivatives, specifically allantoin and uric acid, were determined according to the method described by Chen and Gomes (1992).

### Milk yield and composition

Cows were mechanically milked twice daily at 0600 and 1600 h. Milk yield was recorded daily throughout the experimental period, and mean milk yield was calculated as the average production across all experimental days. Milk yield was corrected to 3.5% fat (MYa) according to NASEM (2021), using the following equation: MYa = (0.432 + 0.163 *fat, %) *milk yield. Milk samples for composition analysis were collected from days 12 to 15 of each experimental period. Samples were obtained from both daily milkings using proportional sampling based on milk yield. Milk fat, protein, and lactose concentrations were determined using a Lactoscan® milk analyzer, which operates based on infrared absorption at specific wavelengths by milk constituents.

### Milk fatty acid composition

For analysis of milk fatty acid composition, approximately 500 mL of milk was collected from each cow. Fatty acids were quantified by gas chromatography (Shimadzu GC-2010, equipped with an automatic injector) using an SP-2560 capillary column (100 m × 0.25 mm internal diameter × 0.20 µm film thickness; Supelco, Bellefonte, PA, USA).

The initial oven temperature was set at 70 °C for 4 min, followed by an increase of 13 °C min^−^^1^ to 175 °C, which was kept for 27 min. Subsequently, temperature was increased at 4 °C min^−^^1^ to 215 °C and held for 31 min. Hydrogen (H₂) was used as the carrier gas at a linear velocity of 40 cm s^−^^1^ during chromatographic separation.

Four standards were used for fatty acid identification: a C4–C24 fatty acid methyl ester standard mixture (Supelco® 37 Component FAME Mix), vaccenic acid (C18:1 trans-11; V038-1 g, Sigma®), conjugated linoleic acid (CLA) C18:2 trans-10, cis-12 (UC-61 M, 100 mg), and C18:2 cis-9, trans-11 (UC-60 M, 100 mg; NU-CHEK-PREP, USA). These standards were used to identify fatty acids formed during ruminal biohydrogenation of unsaturated fatty acids.

### Calculations and statistical analysis

Data were analyzed using SAS OnDemand for Academics (SAS Institute Inc., Cary, NC, USA) (https://www.sas.com/pt_br/software/on-demand-for-academics.html). Residual normality and homogeneity of variances were verified using PROC UNIVARIATE, adopting a significance level of 5% (α = 0.05). Data were analyzed using PROC MIXED according to the following model:

Yijkl = μ + Ti+Aj+ Pk + eijkl,

Wherein: Yijkl = dependent variable, μ = overall mean, Ti = fixed effect of MDSM inclusion level (i = 1–5), Aj = random effect of animal (j = 1–10), Pk = random effect of period (k = 1–5), and eijkl = residual error.

Latin square effects nested within treatment were considered as the whole-plot error term. Degrees of freedom were adjusted using the Kenward–Roger method (DDFM = KR). Effects of increasing dietary inclusion of MDSM were evaluated using polynomial contrasts to evaluate linear and quadratic responses.

To assess relationships among variables and determine their relative contribution to sample characterization, multivariate statistical analysis was performed using principal component analysis (PCA) with PAST® software (version 4.03; Hammer et al., 2001).

## Results

### Particle selection and feed intake

Natural matter and dry matter contents of fecal feed residues were not affected (*p* > 0.05) by the experimental diets (Table 2). Particle distribution measured using the Penn State Particle Separator differed among diets and feed refusals. Diets differed (*p* < 0.05) in particle selectivity for sieves with openings of 38–19 mm and 19–8 mm compared with the control diet.

**Table 2:** Fecal feed residues, particle size distribution, and selection index according to experimental diets

Item	Micronized defatted soybean meal (% DM basis)					SEM^1^	p-value^2^		
	0	25	50	75	100		Diet	Linear	Quad
Fecal feed waste (%)									
Natural matter	26.80	30.70	28.20	25.60	25.20	1.41	0.58	0.33	0.38
Dry matter	25.60	28.10	23.70	21.60	23.00	1.79	0.37	0.14	0.99
Diet particle size distribution (%)									
>19 mm	28.40	26.70	27.70	28.10	26.50	2.03	0.99	0.78	0.80
8–19 mm	23.6c	30.5a	29.4ab	31.4a	27.3b	1.45	0.00	0.02	0.01
4–8 mm	6.5c	12.1a	10.9b	9.5b	10.7b	1.09	0.04	0.29	0.04
<4 mm	41.30	31.60	31.50	32.80	35.80	2.06	0.46	0.66	0.28
Refusals retained on particle separator (%)									
>19 mm	36.3b	39.8a	44.1a	29.4c	25.1c	3.78	0.04	0.07	0.02
8–19 mm	32.10	31.90	29.30	31.30	38.90	1.95	0.34	0.22	0.14
4–8 mm	5.30	9.60	7.20	8.70	7.00	0.62	0.07	0.20	0.10
<4 mm	25.9b	18.0c	18.6c	29.3a	27.2ab	2.66	0.03	0.06	0.11
Selection index^4^									
>19 mm	1.06	1.00	1.03	1.05	1.01	0.03	0.93	0.93	0.92
8–19 mm	1.04	1.03	1.08	1.06	1.00	0.04	0.82	0.79	0.35
4–8 mm	1.08	1.05	1.10	1.08	1.03	0.03	0.49	0.28	0.95
<4 mm	1.01	1.07	1.11	1.08	1.04	0.03	0.90	0.51	0.73

Means followed by the same letter within a row do not differ according to Tukey’s test (*p* < 0.05).

^1^SEM^:^ standard error of the mean; ^2^ *p*-value: Probability of dietary treatment effect; Lin: linear effect; Quad: quadratic effect; ^4^Particle selection index calculated according to Leonardi and Armentano (2003). Values below 1 indicate particle size rejection, while values above 1 indicate particle size selection

Compared to the control diet, the proportion of refusals retained on the >38-mm sieve decreased (*p* < 0.05) by 30.97% in the diet containing 100% MDSM inclusion, showing both linear and quadratic effects (*p* < 0.053). Conversely, particles retained on the 19–8 mm sieve increased (*p* < 0.05) by 67.69% in the diet containing 50% MDSM inclusion, with a tendency toward a quadratic response. Selection indices were not affected (*p* > 0.05) by the experimental diets.

### Dry matter and nutrient intake and digestibility

No differences among experimental diets were observed for dry matter intake (mean = 14.58 kg day^−^^1^; *p* = 0.08), organic matter intake (mean = 13.60 kg day^−^^1^; *p* = 0.08), starch intake (mean = 4.26 kg day^−^^1^; *p* = 0.12), or neutral detergent fiber (NDF) intake (mean = 5.04 kg day^−^^1^; *p* = 0.07).

Increasing dietary inclusion of MDSM affected crude protein intake, which followed a quadratic response with a maximum estimated at 58% MDSM inclusion (Table 3). Ether extract intake decreased linearly as MDSM inclusion increased, with a reduction of 0.0225% for each 1% increase in dietary MDSM.

**Table 3:** Nutrient intake and digestibility of lactating Jersey cows fed diets containing increasing proportions of micronized defatted soybean meal replacing whole soybean grain

Item	Micronized defatted soybean meal (% DM basis)^1^					**SEM**^**2**^	**p-value**^**3**^		
	0	25	50	75	100		Diet	Linear	Quad
*Intake (*kg day^−1^)									
Dry matter	14.50	14.50	14.30	15.00	14.60	0.48	0.08	0.61	0.34
Organic matter	13.30	14.40	13.20	13.80	13.30	0.44	0.08	0.58	0.30
Crude protein	2.20	2.50	2.55	2.50	2.40	0.07	0.05	0.07	0.01
Ether extract	1.06	0.96	0.78	0.67	0.53	0.04	<0.01	<0.01	0.69
Starch	4.20	4.50	4.10	4.30	4.20	0.13	0.12	0.37	0.40
Neutral detergent fiber	5.00	5.40	4.80	5.10	4.90	0.18	0.07	0.33	0.28
Nonfiber carbohydrates	5.0c	5.5a	5.2c	5.4b	5.4b	0.17	0.04	0.05	0.37
Total digestible nutrients	9.8c	10.4a	9.4c	9.6b	9.2b	0.31	0.01	0.01	0.33
Net energy for lactation (Mcal day^−1^)	40.70	43.20	39.40	40.70	39.00	1.31	0.06	0.07	0.37
*Intake (% BW)*									
Dry matter	3.40	3.70	3.60	3.60	3.70	0.11	0.51	0.36	0.48
Neutral detergent fiber	1.10	1.30	1.30	1.30	1.30	0.04	0.06	0.01	0.49
*Digestibility (g kg*^*-1*^)									
Dry matter	617.3	674.1	685.9	691.8	693.0	13.77	<0.01	<0.01	0.77
Organic matter	636.2	691.6	699.4	707.4	715.0	13.14	<0.01	<0.01	0.92
Crude protein	667.8	686.6	714.8	717.0	719.6	14.24	0.00	0.01	0.77
Ether extract	907.6	777.5	829.3	854.9	876.6	15.76	0.03	0.04	0.64
Starch	899.0	920.7	923.3	936.5	939.5	8.55	<0.01	<0.01	0.88
Neutral detergent fiber	581.20	656.70	661.20	545.00	653.20	22.47	0.18	0.80	0.71

Means followed by the same letter within a row do not differ according to Tukey’s test (*p* < 0.05)

^1^ Inclusion levels of micronized defatted soybean meal (% in total mixed diet)

^2^ SEM - standard error of the mean

^3^ *p*-value- Probability of dietary treatment effect; Lin (linear effect); Quad (quadratic effect)

Intake of Nonfiber carbohydrates, total digestible nutrients, and net energy for lactation showed quadratic responses to MDSM inclusion. No differences were observed among diets for dry matter intake (*p* = 0.51) or NDF intake (*p* = 0.06) when expressed as percentage of body weight.

Replacement of whole soybean grain with MDSM altered digestibility of dry matter and most nutrients, except NDF (*p* = 0.18). Increasing MDSM inclusion resulted in linear increases (*p* < 0.05) in digestibility of dry matter, organic matter, crude protein, ether extract, and starch (Table 3).

### Ruminal parameters

Experimental diets containing MDSM did not affect ruminal pH (*p* = 0.87), which averaged 6.76 (Table 4). Increasing dietary MDSM inclusion influenced concentrations of acetic and propionic acids, both showing quadratic responses with estimated maxima at 50.7% and 40.8% MDSM inclusion, respectively.

**Table 4:** Ruminal parameters of lactating Jersey cows fed diets containing increasing proportions of micronized defatted soybean meal replacing whole soybean grain

Item	Micronized defatted soybean meal (% DM basis)^1^					**SEM**^**2**^	**p-value**^**3**^		
	0	25	50	75	100		Diet	Linear	Quad
pH	6.80	6.70	6.90	6.70	6.70	0.07	0.87	0.62	0.51
*Volatile fatty acids (mmol L*^*-1*^)									
Acetate	56.2	60.1	60.6	62.6	51.9	2.69	0.05	0.55	0.01
Propionate	20.4	19.5	19.3	22.4	17.8	0.99	0.05	0.37	0.00
Butyrate	11.70	10.00	9.80	10.60	8.90	0.67	0.74	0.30	0.86
Isobutyrate	0.91	0.78	0.79	0.75	0.82	0.05	0.87	0.55	0.42
Valerate	2.28	0.98	1.00	1.17	1.10	0.20	0.15	0.12	0.08
Isovalerate	1.00	1.20	1.20	1.30	1.00	0.08	0.76	0.79	0.22
Total	96.8	95.6	95.9	102.2	84.8	4.37	0.03	0.97	0.02
Acetate: propionate ratio	2.70	3.30	3.10	2.80	2.90	0.08	0.39	0.70	0.16
*Microbial protein synthesis (g day*^-^)^*1*^									
Microbial nitrogen	67.3	49.9	52.1	49.5	57.3	4.78	0.67	0.51	0.23
Microbial protein	421	312	326	309.9	358.6	2.99	0.67	0.51	0.23

^1^ Inclusion levels of micronized defatted soybean meal (% in total mixed diet)

^2^ SEM - standard error of the mean

^3^ *p*-value- Probability of dietary treatment effect; Lin (linear effect); Quad (quadratic effect)

Total volatile fatty acid concentration also exhibited a quadratic response, with maximum production estimated at 38.4% MDSM inclusion. Microbial protein synthesis was not affected by dietary treatments (*p* = 0.67), averaging 345.5 g day^−^^1^.

### Milk yield and composition

Partial replacement of whole soybean grain with MDSM did not affect milk yield (*p* = 0.12; mean = 14.84 kg day^−^^1^) or overall milk composition (Table 5). However, milk yield corrected to 3.5% fat increased linearly as dietary MDSM inclusion increased.

**Table 5:** Milk yield and physicochemical composition of milk from lactating Jersey cows fed diets containing increasing proportions of micronized defatted soybean meal replacing whole soybean grain

Item	Micronized defatted soybean meal (% DM basis)^1^					SEM^2^	*p*-value^3^		
	0	25	50	75	100		Diet	Linear	Quad
	kg day^−1^								
Milk yield	14.87	13.81	15.24	14.06	16.24	0.39	0.12	0.17	0.13
Milk yield corrected to 3.5% fat (3.5% FCM)	17.92b	15.81c	19.11a	16.04b	19.65a	0.54	0.01	0.01	0.10
Fat	0.707	0.606	0.770	0.613	0.777	0.03	0.30	0.29	0.20
Protein	0.46	0.44	0.48	0.44	0.50	0.01	0.22	0.27	0.28
Lactose	0.69	0.66	0.72	0.66	0.75	0.02	0.21	0.26	0.27
	Percentage (%)								
Fat	4.73	4.43	5.08	4.33	4.83	0.14	0.41	0.91	0.87
Protein	3.12	3.17	3.14	3.13	3.07	0.03	0.86	0.49	0.43
Lactose	4.67	4.74	4.70	4.69	4.60	0.05	0.88	0.51	0.45
	Feed Efficiency								
MY/DMI^4^	1.03	0.874	1.08	0.950	1.15	0.04	0.30	0.10	0.08
FCM/DMI^5^	1.20a	0.982b	1.31a	1.05b	1.33a	0.05	0.01	0.01	0.13

Means followed by the same letter within a row do not differ according to Tukey’s test (*p* < 0.05)

^1^Micronized defatted soybean meal (% of total animal diet)

^2^SEM - standard error of the mean

^3^ Probability of dietary treatment effect; Lin (linear effect); Quad (quadratic effect)

^4^ Feed efficiency (MY/DMI) = Milk yield (kg day^−1^) divided by dry matter intake (kg day^−1^)

^5^ Feed efficiency (3.5% FCM/DMI) = milk yield corrected to 3.5% fat (kg day^−^^1^) divided by dry matter intake (kg day^−^^1^)

Milk fat (*p* = 0.41), protein (*p* = 0.86), and lactose (*p* = 0.88) concentrations were not affected by treatment, averaging 4.68%, 3.13%, and 4.68%, respectively. Feed efficiency calculated from milk yield did not differ among treatments; however, the diet containing 100% MDSM numerically maximized feed efficiency.

### Milk fatty acid profile

Dietary treatments affected concentrations of C6:0 and C17:0 fatty acids in milk (Table 6). For each 1% increase in dietary MDSM inclusion, concentrations increased by 0.0022 and 0.0005 g 100 g^−^^1^ of milk fatty acids, respectively.

**Table 6:** Milk fatty acid profile of lactating Jersey cows fed diets containing increasing proportions of micronized defatted soybean meal replacing whole soybean grain

Fatty acids (g/100 g)	Micronized defatted soybean meal (% DM basis)^1^					**SEM**^**2**^	**p-value**^**3**^		
	0	25	50	75	100		Diet	Linear	Quad
C4:0	1.57	1.58	1.58	1.59	1.58	0.00	0.95	0.58	0.87
C6:0	1.65b	1.63b	1.67a	1.66a	1.70a	0.00	0.05	0.04	0.32
C8:0	2.94	2.94	2.86	2.93	2.91	0.00	0.54	0.45	0.43
C10:0	6.59	6.49	6.75	6.79	6.65	0.13	0.11	0.12	0.31
C12:0	4.21	4.21	4.23	4.19	4.21	0.07	0.60	0.68	0.95
C14:0	11.16	11.09	11.07	11.08	10.89	0.34	0.42	0.10	0.58
C14:1	0.05	0.05	0.05	0.05	0.05	0.00	0.74	0.36	0.43
C15:0	1.43	1.50	1.47	1.46	1.46	0.00	0.18	0.71	0.12
C15:1	0.19	0.20	0.20	0.20	0.20	0.00	0.68	0.45	0.52
C16:0	27.94	27.38	27.71	27.07	27.82	2.45	0.30	0.57	0.17
C16:1	0.94	0.95	0.94	0.97	0.97	0.00	0.57	0.18	0.97
C17:0	0.160c	0.182a	0.176b	0.178a	0.178a	0.00	0.05	0.05	0.07
C17:1	0.47	0.41	0.44	0.43	0.44	0.00	0.07	0.29	0.06
C18:0	14.47	14.30	14.29	14.39	14.27	1.54	0.45	0.25	0.52
*cis 11*,C18:1	7.02c	7.10b	7.18a	7.25a	7.12b	2.44	0.01	0.55	0.01
*cis 9*,C18:1	13.11c	13.51a	13.37a	13.29b	13.28b	1.44	0.03	0.65	0.02
cis-9,cis-12 C18:2	1.59	2.01	1.58	2.01	1.76	0.03	0.21	0.52	0.46
cis-9,cis-12,cis-15 C18:3	1.60	1.59	1.57	1.61	1.63	0.00	0.66	0.40	0.31
cis-9,trans-11 CLA	0.87	0.87	0.85	0.87	0.86	0.00	0.75	0.99	0.63
C20:0	0.90	0.90	0.93	0.89	0.94	0.00	0.47	0.36	0.66
C22:0	0.85	0.86	0.86	0.85	0.85	0.00	0.84	0.44	0.46
C24:0	0.23	0.21	0.19	0.20	0.20	0.00	0.28	0.15	0.15
Total									
Ʃ 4–14C^4^	28.20	28.01	28.23	28.30	28.00	1.23	0.33	0.81	0.43
Ʃ above 16C^5^	70.17	70.28	70.10	70.03	70.33	2.56	0.45	0.89	0.31
Ʃ SFA^6^	74.14	73.29	73.80	73.30	73.68	3.33	0.41	0.42	0.29
Ʃ UFA^7^	25.85	26.70	26.19	26.69	26.32	1.44	0.41	0.42	0.29
Ʃ MCFA^8^	21.78	22.22	22.18	22.19	22.05	2.02	0.51	0.42	0.15
Ʃ SCFA^9^	4.06	4.47	4.00	4.50	4.26	0.03	0.24	0.47	0.63
Ʃ LCFA^10^	2.25	2.29	2.27	2.26	2.27	0.03	0.93	0.86	0.63
SFA:UFA ratio^11^	2.86	2.75	2.81	2.74	2.80	0.01	0.42	0.39	0.29
C18 SFA:C18 UFA^12^	0.60	0.57	0.58	0.58	0.58	0.00	0.73	0.29	0.26
Product-to-substrate ratio^11^									
*cis*-9 C14:1-C14:0	0.00	0.01	0.01	0.01	0.01	0.00	0.67	0.21	0.53
*cis*-9 C16:1-C16:0	0.03	0.03	0.03	0.04	0.03	0.00	0.42	0.26	0.49
*cis*-9 C18:1-C18:0	1.39	1.44	1.43	1.42	1.42	0.03	0.35	0.29	0.15

Means followed by the same letter within a row do not differ according to Tukey’s test (*p* < 0.05)

^1^ Micronized defatted soybean meal (% of total animal diet)

^2^ SEM - standard error of the mean

^3^ Probability of dietary treatment effect; Lin (linear effect); Quad (quadratic effect)

^4^ Sum of fatty acids with 4 to 14C

^5^ Total fatty acids above 16C

^6^ Total saturated fatty acids

^7^ Total unsaturated fatty acids

^8^ Sum of medium-chain unsaturated fatty acids

^9^ Sum of short-chain unsaturated fatty acids

^10^ Sum of long-chain unsaturated fatty acids

^11^ Saturated: unsaturated fatty acid ratio

^12^ C18 saturated:C18 unsaturated fatty acid ratio

Inclusion of MDSM increased concentrations of long-chain fatty acids cis-11 C18:1 and cis-9 C18:1, which followed quadratic responses with estimated maxima at 60.9 and 53% MDSM inclusion (DM basis), respectively. No differences were observed among treatments for other milk fatty acid variables.

**Fig. 1:**
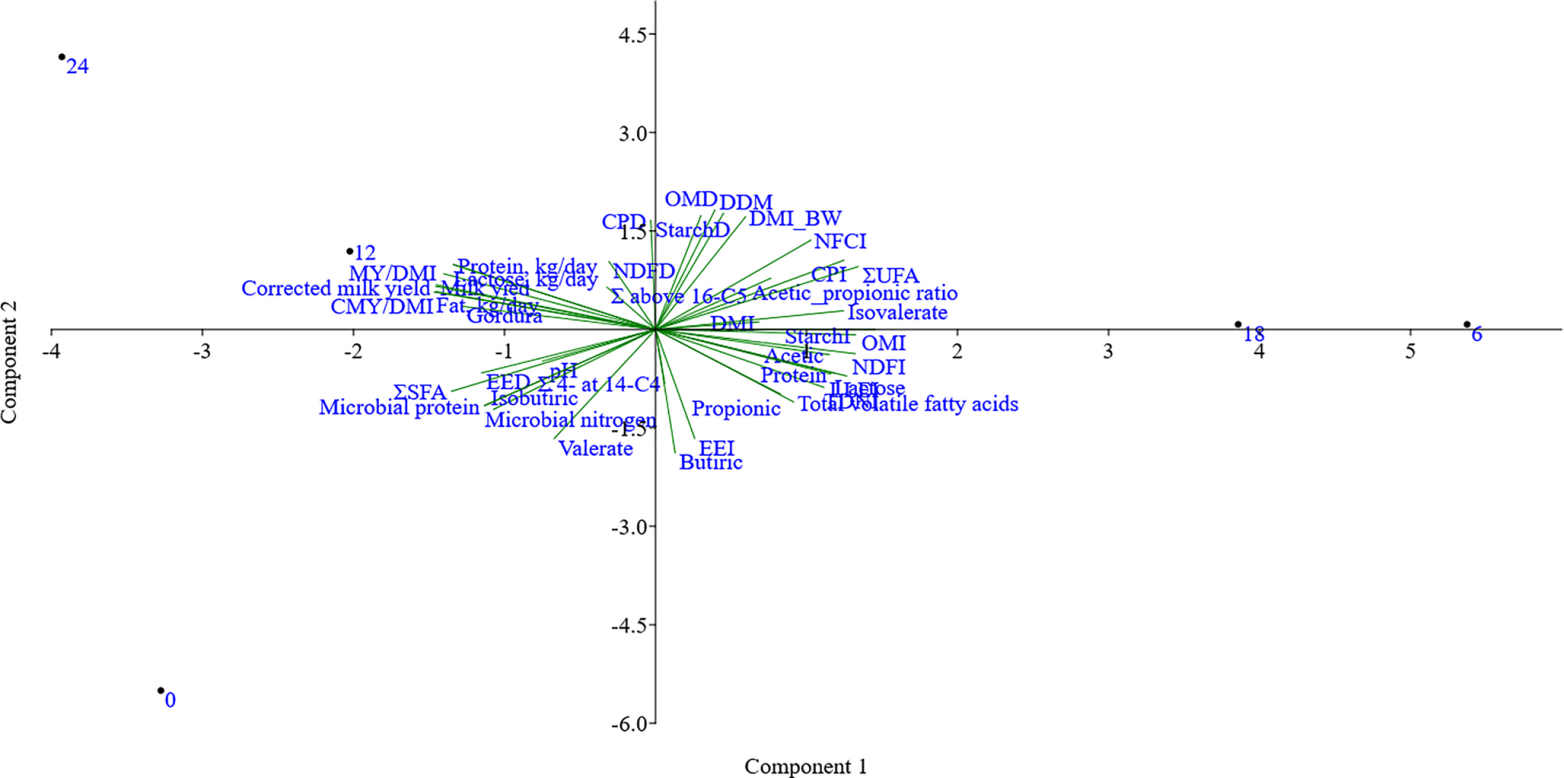
Schematic representation of the first (CP1) and second (CP2) principal components from the analysis of the evaluated variables

Principal component analysis (PCA) indicated that PC1 and PC2 together explained 74.99% of total data variation, with PC1 accounting for 45.14% (Fig. 1). Isolated dependent variables with the greatest loadings in PC1 included organic matter intake (0.2151), sum of unsaturated fatty acids (0.1985), starch intake (0.1959), and crude protein intake (0.1844). Dependent variables with the highest eigenvector coefficients in both PC1 and PC2 were crude protein intake (0.1844 and 0.1561), Nonfiber carbohydrate intake (0.1521 and 0.2012), and dry matter intake expressed as percentage of body weight (0.0881 and 0.2540).

## Discussion

Whole raw soybeans are feedstuffs rich in crude protein (38.47%) and ether extract (18.91%). Their inclusion in diets for lactating cows offers several advantages, including operational benefits associated with the absence of processing steps such as grinding or heat treatment, which reduces labor and energy costs. However, the hull (pericarp) of whole soybeans may limit ruminal nutrient digestibility, in addition to potential negative effects associated with antinutritional factors.

Micronized defatted soybean meal (MDSM) represents a protein source for both ruminal microorganisms and the host animal, with potential to replace whole soybeans due to increased nutrient availability, as observed in the present study. Moreover, dry matter intake (DMI) and neutral detergent fiber (NDF) intake were not affected by replacement of whole soybean grain with MDSM, averaging 14.58 and 5.04 kg day^−^^1^, respectively. These values are consistent with requirements predicted by NASEM (2021) and Leal et al. (2023) for the observed level of milk production.

A higher crude protein (CP) intake was observed when 58.33% of whole soybean grain was replaced with micronized defatted soybean meal (MDSM) in the diet. Crude protein intake is essential because it supplies rumen-degradable protein that is converted into ammonia and subsequently used for microbial protein synthesis in the presence of available carbon skeletons. In addition, dietary CP provides amino acids that are absorbed in the small intestine and utilized for physiological functions related to maintenance, production, and reproduction.

From a production perspective, increased CP intake is desirable only when it results in greater milk yield, indicating improved efficiency of protein utilization. Elevated CP intake without corresponding improvements in productivity suggests nitrogen losses through feces and urine (Santana et al. 2019, Santana et al. 2019). Supplemental dietary protein may be provided to minimize the risk of amino acid deficiencies at the intestinal level, which could otherwise reduce lactational performance (Reynal and Broderick 2003).

In the present study, replacement of approximately 50% of whole soybean grain with MDSM was not advantageous, as microbial protein synthesis and milk yield (mean = 14.84 kg day^−^^1^) were not affected. However, cows fed diets containing 100% MDSM produced greater fat-corrected milk yield while maintaining similar dry matter intake compared with other treatments, indicating improved nutrient-use efficiency.

These findings are supported by the multivariate analysis, in which CP intake was identified as one of the dependent variables with the highest loadings in both PC1 and PC2, which together explained more than 70% of total data variation. This result is particularly relevant because CP is typically the most expensive nutrient component in dairy cow diets. Excess dietary protein increases feeding costs, reduces nutrient-use efficiency (Soares et al. 2020; Marques et al. 2024), and may negatively affect reproductive performance (Rajala-Schultz et al. 2001).

Principal component analysis (PCA) is a statistical technique primarily used to reduce dimensionality of complex datasets while retaining most of the original variability. Its application deals with large data amounts and facilitates identification of key variables driving treatment responses.

Replacement of whole soybean grain with micronized defatted soybean meal (MDSM) in diets for Jersey cows increased digestibility of dry matter, organic matter, crude protein, and starch, which likely contributed to the greater milk yield corrected to 4% fat observed in this study. According to multivariate regression estimates reported by NRC, National (2001), milk yield increases by approximately 0.75 kg day^−^^1^ when dietary crude protein (CP) concentration rises from 15 to 16% and by 0.35 kg day^−^^1^ when CP increases from 19 to 20%. Consistent with these observations, the higher CP intake—approximately 200 g day^−^^1^ greater in cows receiving the diet containing 100% MDSM compared with the control diet—was associated with increased milk yield.

Imaizumi et al. (2010) reported that higher dry matter intake associated with increased dietary CP may enhance ruminal fermentation activity, resulting in greater production of short-chain fatty acids. In the present study, increased fermentative activity was observed in cows fed diets containing 75% MDSM, which showed greater total short-chain fatty acid production compared with other treatments. Cows receiving the 75% MDSM diet also exhibited higher ruminal acetate and propionate production; however, these changes were not reflected in milk yield or milk composition.

Regarding the milk fatty acid profile, inclusion of MDSM in diets for Jersey cows did not affect concentrations of linoleic acid (C18:2) or linolenic acid (C18:3) in milk fat. According to Lock and Bauman (2004), dietary vegetable oils such as soybean products can increase conjugated linoleic acid (CLA) content in milk fat because of their high linoleic acid (C18:2) concentration. These authors also reported that synthesis of linoleic- and linolenic-derived fatty acids in lactating cows occurs through both ruminal and tissue pathways.

In the rumen, linolenic acid (cis-9, cis-12, cis-15 C18:3) undergoes biohydrogenation, forming intermediates such as cis-9, trans-11, cis-15 C18:3 and trans-11, cis-15 C18:2, which are subsequently converted to trans-11 C18:1 (vaccenic acid). In body tissues, vaccenic acid is desaturated by the Δ9-desaturase enzyme to form cis-9, trans-11 CLA. Similarly, linoleic acid (cis-9, cis-12 C18:2) is partially converted in the rumen to cis-9, trans-11 CLA, which can then be absorbed and further metabolized in tissues. Depending on the dietary fatty acid source, ruminal biohydrogenation may modify fatty acid structures, thereby altering the milk fat profile.

Barletta et al. (2016) suggested that whole raw soybeans may provide partial protection against ruminal biohydrogenation, potentially affecting microbial activity in the rumen. Formation of specific CLA isomers, particularly trans-10, cis-12 CLA, may negatively influence milk fat concentration, although CLA is also associated with beneficial nutraceutical properties, including anti-carcinogenic and anti-atherogenic effects (Lock and Bauman 2004; Costa et al. 2020). Increased CLA concentration therefore enhances the functional value of milk.

CLA present in milk fat originates partly from ruminal biohydrogenation of linoleic acid and partly from Δ9-desaturase activity in mammary gland cells, which converts absorbed vaccenic acid into CLA (Bauman and Griinari, 2001). In the present study, reduced ether extract intake in cows receiving higher proportions of MDSM did not alter concentrations of saturated or unsaturated fatty acids in milk fat.

Total replacement of whole soybean grain with MDSM in diets for Jersey cows increased milk yield, improved feed efficiency, and did not alter milk composition. In addition, MDSM inclusion enhanced digestibility of dry matter, organic matter, crude protein, and starch.

Higher dietary inclusion of MDSM also modified the profile of long-chain fatty acids in milk fat, particularly cis-11 C18:1 and cis-9 C18:1.

## Data Availability

The data supporting the findings of this study are available from the corresponding authors upon reasonable request.
